# Topology-Aware Lane Detection with Relational Reasoning and Consistency Constraints

**DOI:** 10.3390/s26134278

**Published:** 2026-07-05

**Authors:** Danyang Dong, Qibo Zhang, Yihui Zhan, Tianqing Su, Quanke Su, Samuel S. Mao, Yusheng Xiang

**Affiliations:** 1 Center for Intelligent Autonomous Systems, Nantong University, Nantong 226019, China; danyangdong@stmail.ntu.edu.cn (D.D.); qibo.zhang@stmail.ntu.edu.cn (Q.Z.); zyhntu@stmail.ntu.edu.cn (Y.Z.); 2 Department of AI, SunnyWay LLC, Suzhou 215000, China; 3 Thrust of Intelligent Transportation, The Hong Kong University of Science and Technology (Guangzhou), Guangzhou 511458, China; quankesu@hkust-gz.edu.cn; 4 Department of Mechanical Engineering, University of California at Berkeley, Berkeley, CA 94720, USA; ssmao@berkeley.edu

**Keywords:** lane detection, topology-aware learning, autonomous driving, deep learning, structural consistency

## Abstract

Lane detection is a fundamental perception task for autonomous driving and intelligent transportation systems. Although existing methods have achieved promising performance, many of them mainly focus on individual lane instances and insufficiently exploit the structural relationships among lanes, such as relative ordering, geometric continuity, and spatial parallelism. This limitation may lead to broken lanes, ordering errors, and geometric inconsistencies in complex road scenarios. To address these issues, we propose TPDNet, a topology-aware lane detection framework that incorporates structural reasoning into the detection pipeline at three complementary levels. First, a Topology-aware Perception Reasoner (TPR) is introduced at the feature level to capture relational dependencies among lane features and enhance the representation of global road topology. Second, a Topology-Decoupled Head (TDH) is designed at the prediction level to decouple geometric regression from lane classification, thereby reducing task interference and improving prediction stability. Third, a Topology Consistency Loss (TCL) is formulated as a complementary supervision term to encourage smoothness and ordering consistency in predicted lanes. Extensive experiments on three public benchmarks demonstrate the effectiveness of the proposed method. On CULane, TPDNet achieves an F1@50 of 81.46 with ResNet101 and remains competitive with the strongest compared methods, while showing improved robustness in challenging scenarios such as Curve and Dazzle light. On TuSimple, TPDNet obtains an F1 score of 98.01 among the compared methods, while maintaining competitive accuracy. On CurveLanes, TPDNet achieves an mF1 of 58.74, exceeding the strongest baseline by 4.98 points. These results suggest that topology-aware reasoning can improve the generalization capability of lane detection and help produce more structurally coherent lane predictions under diverse road conditions.

## 1. Introduction

**Figure 1:**
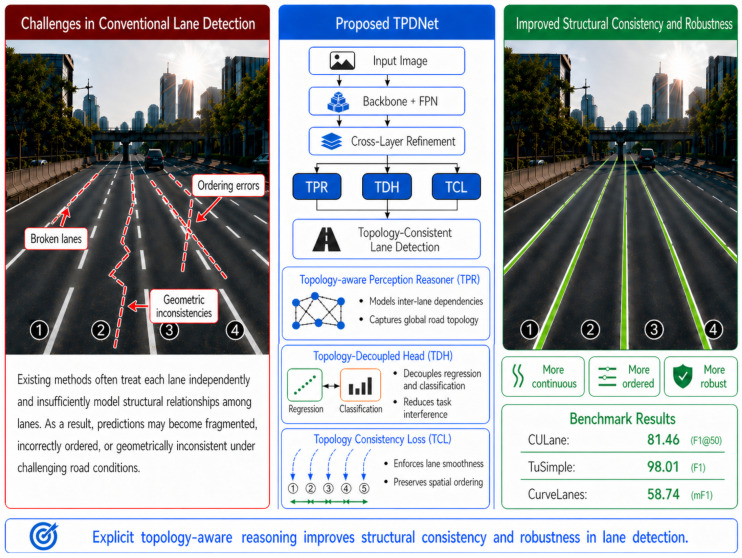
Motivation and conceptual overview of TPDNet. Existing lane detection methods may suffer from broken lanes, ordering errors, and geometric inconsistencies under challenging road conditions. TPDNet introduces topology-aware reasoning at the feature, prediction, and supervision levels through TPR, TDH, and TCL, respectively, producing more continuous, ordered, and robust lane predictions.

Lane detection is a fundamental perception task for intelligent transportation systems [1,2] and autonomous driving, providing essential structural cues for vehicle localization, path planning, and driving safety. Accurate and robust lane detection remains challenging due to the intrinsic properties of lane markings, which are typically thin, elongated, and distributed across the entire image. In real-world driving scenarios, lane lines are often affected by occlusion, illumination variations, worn markings, and complex road geometries, making reliable perception particularly difficult. As illustrated in Figure 1, these challenges may lead to broken lane predictions, ordering ambiguities, and geometric inconsistencies, motivating the topology-aware design of TPDNet.

Early deep learning-based approaches predominantly formulate lane detection as a pixel-wise segmentation problem [3,4,5]. By predicting dense lane masks, segmentation-based methods are capable of flexibly modeling arbitrary lane shapes. To enhance robustness under weak visual cues, representative works introduce spatial message passing or recurrent feature aggregation [5,6] mechanisms to capture long-range contextual information. Despite their effectiveness, these methods usually involve dense pixel-level computation, resulting in high computational cost and limited efficiency [7,8], which hinders real-time deployment in practical systems.

To address these limitations, subsequent studies reformulate lane detection as a structured prediction problem. Among them, anchor-based approaches introduce predefined lane priors and regress lane positions as holistic entities, leveraging strong geometric constraints to improve stability and efficiency [7,8,9,10,11]. Early anchor-based methods demonstrate that incorporating global contextual information into anchor representations significantly improves robustness under occlusion. More recent works further enhance this paradigm by exploiting cross-layer refinement strategies, global context aggregation, and lane-aware loss functions, enabling better integration of high-level semantic information and low-level geometric details. These advances have shown strong performance in challenging scenarios such as dense lanes, curved structures, and complex topologies, while maintaining favorable real-time performance.

In parallel, inspired by the success of Transformer architectures in computer vision [12,13], attention-based lane detection methods have been proposed [14,15,16] to explicitly model long-range dependencies. By leveraging self-attention mechanisms, these approaches exhibit strong capabilities in capturing lane continuity and contextual relationships [14,16], particularly in scenarios with sparse or ambiguous visual evidence. Existing Transformer-style designs for lane detection can be roughly divided into image-token attention, which models dense spatial interactions in the feature map, and lane-query attention, which updates a set of lane proposals or queries. The former provides global context but often involves redundant background interactions, whereas the latter is more efficient but may still treat lane queries mainly as appearance-driven tokens. Several studies further introduce query-based or multi-granularity attention mechanisms to reduce redundancy and emphasize informative regions. However, existing attention-driven methods often rely on dense global attention or generic query similarity, which may weaken the focus on fine-grained geometric localization and explicit inter-lane topology. Effectively combining global relational reasoning with precise local structure modeling therefore remains a non-trivial challenge.

Despite these advances, many existing methods still primarily optimize lane predictions at the instance or point level [17,18], while the explicit modeling of inter-lane structural relationships remains insufficient. In real road scenes, lane markings are not isolated visual elements, but structured components of an organized road topology. Adjacent lanes usually maintain consistent relative ordering, lane trajectories exhibit smooth geometric continuity, and multiple lanes often follow spatially coherent layouts such as parallelism or gradual convergence. When such structural patterns are not explicitly encoded, lane predictions may become fragmented, incorrectly ordered, or geometrically inconsistent, particularly under unreliable visual cues such as occlusion, shadows, worn markings, curved roads, and Dazzle light.

To address these issues, we propose TPDNet, a topology-aware lane detection framework that explicitly incorporates structural reasoning into the lane detection pipeline. Rather than correcting inconsistent predictions through post-processing, TPDNet models lane topology at three complementary levels: feature representation, prediction decoupling, and supervised optimization. At the feature level, we introduce a Topology-aware Perception Reasoner (TPR) to capture relational dependencies among lane representations, enabling the network to reason about global road structure rather than processing each lane in isolation. At the prediction level, a Topology-Decoupled Head (TDH) disentangles geometric regression from lane classification, alleviating task interference and improving localization stability. At the supervision level, a Topology Consistency Loss (TCL) imposes smoothness and ordering regularization on predicted lanes, guiding the model toward structurally coherent outputs under challenging conditions.

Extensive experiments on CULane [5], TuSimple, and CurveLanes [19] validate the effectiveness of TPDNet. On CULane, our ResNet101 variant achieves an F1@50 of 81.46, with improved robustness in challenging scenarios such as Curve and Dazzle light. On TuSimple, TPDNet obtains an F1 score of 98.01 among the compared methods, while maintaining competitive accuracy under the same evaluation protocol. On CurveLanes, TPDNet achieves an mF1 of 58.74, exceeding the strongest baseline by 4.98 points. These results demonstrate that topology-aware reasoning improves lane detection accuracy and supports more structurally coherent predictions across diverse road conditions.

The main contributions of this work are summarized as follows:

- We revisit lane detection from a structural topology perspective and identify insufficient inter-lane relationship modeling as a key limitation of existing lane detection pipelines. Based on this observation, we propose TPDNet, a unified topology-aware framework that incorporates structural reasoning at the feature, prediction, and supervision levels.
- We introduce a Topology-aware Perception Reasoner (TPR) to capture relational dependencies among lane representations, allowing the network to reason about global road structure rather than treating lanes as isolated prediction targets.
- We design a Topology-Decoupled Head (TDH) to disentangle geometric regression from lane classification, thereby alleviating task interference and improving localization stability.
- We formulate a Topology Consistency Loss (TCL) as a complementary structural regularizer that imposes smoothness and ordering constraints on predicted lanes, encouraging structurally coherent lane predictions in complex driving scenarios.

## 2. Methodology

### 2.1. Overview

**Figure 2:**
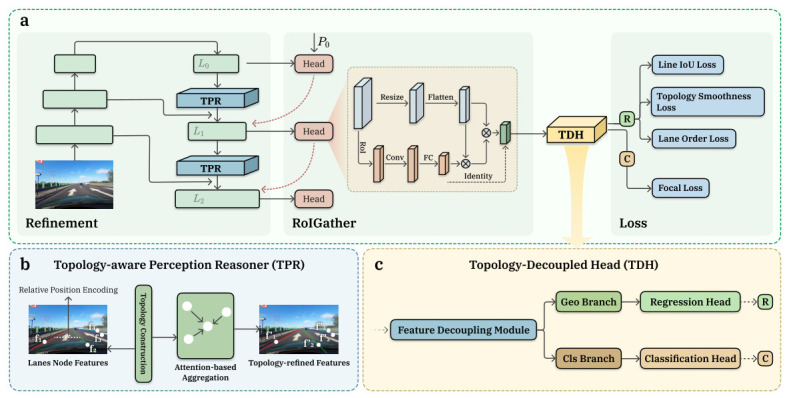
Detailed Architecture of the proposed TPDNet. (**a**) The overall multi-stage refinement framework. Given multi-scale visual features extracted by the backbone and FPN, lane priors are progressively refined through repeated refinement stages. ROIGather enhances lane proposal features with contextual information, while TPR explicitly models inter-lane relational dependencies for topology-aware road-structure reasoning. (**b**) Illustration of TPR, which incorporates relative position encoding and attention-based aggregation to refine lane features according to the structural relationships among candidate lanes. (**c**) Illustration of TDH, which separates geometric regression and lane classification into two branches to alleviate task interference. The network is supervised by Line IoU Loss, Focal Loss, and the proposed topology consistency losses, including topology smoothness loss and lane order loss.

We propose TPDNet, a topology-aware lane detection framework that integrates inter-lane relational reasoning and structural consistency constraints into an iterative lane refinement pipeline. The overall architecture is illustrated in Figure 2. Section 2.2 introduces the lane representation and refinement pipeline, Section 2.3 describes the topology-aware reasoning module, Section 2.4 explains the decoupled prediction head, Section 2.5 presents the structural loss, and Section 2.6 summarizes training and inference. Given an input road image, a ResNet backbone [20] equipped with a Feature Pyramid Network [21] extracts multi-scale visual features. Built on CLRHead [9], TPDNet uses these features to generate initial lane priors, which are then progressively refined through *T* stages.

At each refinement stage, ROI features are first aggregated by the ROIGather module, which enhances each lane proposal with image-level contextual information. The resulting lane-query features are then fed into the TPR, where relational dependencies among candidate lanes are explicitly modeled to capture global road topology. In this way, each lane representation is updated not only according to local visual evidence, but also according to the structural configuration of other lanes.

After the final refinement stage, the topology-enhanced lane features are passed to the TDH. TDH separates geometric regression from lane classification into two independent branches, thereby alleviating task interference and improving prediction stability. During training, the TCL further imposes smoothness and ordering constraints on the predicted lanes, encouraging structurally coherent outputs. Through this design, TPDNet jointly exploits visual appearance, inter-lane topology, and structural supervision for robust lane detection in complex road scenarios.

### 2.2. Lane Representation and Iterative Refinement

Lane markings are thin, elongated, and continuous curvilinear structures. Instead of using bounding-box representations, which are not well suited for describing fine-grained lane geometry, we represent each lane as a structured sequence of *N* equidistantly sampled 2D points along the image height [8,9,22]:

(1) $$P={\left\{\left(x_{n},y_{n}\right)\right\}}_{n=0}^{N-1}.$$

The vertical coordinates are fixed and uniformly sampled along the image height *H*:

(2) $$y_{n}=\frac{H}{N-1}\cdot n,\;n=0,1,\ldots ,N-1.$$

The network predicts the corresponding horizontal coordinates through lateral offsets. Specifically, each lane is initialized from a *Lane Prior* $\tilde{P}={\left\{\left({\tilde{x}}_{n},y_{n}\right)\right\}}_{n=0}^{N-1}$, and the refined horizontal coordinate is obtained as:

(3) $$x_{n}={\tilde{x}}_{n}+\Delta x_{n}.$$

Following the CLRHead formulation [9], each Lane Prior is further parameterized by $\Theta =\{p_{fg},\;l,\;x_{0},\;y_{0},\;\theta \}$, where $p_{fg}$ denotes the foreground probability, *l* the lane length, and $\left(x_{0},y_{0},\theta \right)$ the starting position and initial orientation angle relative to the horizontal axis. This point-based representation provides a compact yet flexible description of lane geometry and is compatible with subsequent iterative refinement.

Given the initial lane priors, TPDNet progressively refines lane geometry through multiple stages. At each stage, ROI features are extracted according to the current lane geometry and used to predict residual updates for the lane parameters. Let ${\left\{L_{t}\right\}}_{t=1}^{T}$ denote the hierarchical feature maps used across the *T* refinement stages, where $L_{1}$ corresponds to the coarsest semantic features and $L_{T}$ corresponds to the finest spatial details. Starting from the initial lane priors $P_{0}$, the lane parameters are iteratively updated as

(4) $$P_{t}=P_{t-1}+R_{t}\left(L_{t},\;P_{t-1}\right),\;t=1,\ldots ,T,$$

where $R_{t}$ denotes the stage-specific refinement function that extracts ROI features according to the previous-stage lane geometry $P_{t-1}$ and predicts residual coordinate updates through fully connected layers [9,23]. This iterative refinement strategy enables coarse lane hypotheses to be gradually adjusted toward accurate lane positions.

To integrate multi-scale visual information, TPDNet adopts a cross-layer refinement strategy. High-level feature maps provide semantic context for identifying lane structures, while low-level feature maps preserve fine-grained spatial details for accurate localization. By progressively aggregating features across different FPN levels, the model can balance global structural integrity and local geometric precision.

At each refinement stage, the extracted ROI features are further enhanced by ROIGather. Specifically, ROIGather aggregates contextual information from the global image feature map and combines it with local lane proposal features through a residual connection. This step enriches each lane proposal with image-level context, which is particularly useful under weak visual cues such as occlusion, shadows, or illumination changes. Let $X_{p}^{\left(t\right)}\in R^{C\times N}$ denote the local ROI features of a lane proposal at stage *t*, and let $X_{f}\in R^{C\times HW}$ denote the flattened global feature map from the FPN. Following the attention mechanism [12], the cross-attention weight matrix $W\in R^{N\times HW}$ is computed as

(5) $$W=\mathrm{softmax}\;\left(\frac{{\left(X_{p}^{\left(t\right)}\right)}^{⊤}X_{f}}{\sqrt{C}}\right).$$

The aggregated global context feature $G^{\left(t\right)}\in R^{C\times N}$ is then obtained by

(6) $$G^{\left(t\right)}=X_{f}\;W^{⊤}.$$

The context-enhanced lane feature is computed through a residual connection with a learnable scalar scaling factor γ:

(7) $${\hat{X}}_{p}^{\left(t\right)}=X_{p}^{\left(t\right)}+\gamma \;G^{\left(t\right)}\;\in R^{C\times N}.$$

The enhanced lane-query features ${\hat{X}}_{p}^{\left(t\right)}$ are then passed to TPR for topology-aware relational reasoning among candidate lanes.

### 2.3. Topology-Aware Perception Reasoner

After ROIGather enriches each lane proposal with image-level contextual information, the resulting lane-query features still need to interact with each other to capture the structural organization of the road. If lane candidates are processed independently, the model may fail to preserve inter-lane relationships such as relative ordering, spatial proximity, parallelism, and geometric continuity. To address this issue, we introduce the TPR, which is applied after ROIGather at each refinement stage to perform topology-aware relational reasoning among all lane candidates.

The key idea of TPR is to treat lane candidates as nodes in a geometry-aware relational graph. Each node represents one lane candidate, and each edge describes the structural dependency between a pair of lanes. Different from standard self-attention, which mainly relies on feature similarity, TPR incorporates both lane-level visual features and pairwise relative geometry to construct topology-aware lane affinities. In this way, each lane representation is updated according to not only its own visual evidence, but also the structural configuration of surrounding lanes.

Compared with previous graph-based lane detectors and generic relational reasoning modules, TPR is distinguished in three aspects. First, the graph nodes are not pixels, anchors, or manually grouped lane segments, but iteratively refined lane candidates whose geometry is updated at each refinement stage. The relational graph is therefore dynamic and evolves with the predicted lane layout. Second, the edge representation is not derived only from feature similarity or Euclidean proximity. It explicitly encodes lane-specific topology cues, including relative start position, orientation, length, shared valid support, mean lateral displacement, and top/bottom separation. Third, the relative geometry affects both the attention logits and the aggregated messages: the bias term changes which lanes communicate, whereas the relation message changes what geometric information is propagated. This dual use is intended to make the reasoning process sensitive to lane ordering, parallelism, convergence, and crossing tendency, rather than merely adding a generic positional encoding to self-attention.

For clarity, the following formulation is written for a single image in a mini-batch, and the batch dimension is omitted. Let *M* denote the number of candidate lanes, *N* denote the number of sampled lateral coordinates for each lane, and *D* denote the dimension of the lane-level feature. TPR uses $H_{a}$ attention heads, and the feature dimension of each head is $d=D/H_{a}$. The superscript (t) denotes the refinement stage. When no ambiguity arises, the stage superscript is omitted in the attention formulation for compactness.

**Lane-level Feature Preparation:** At refinement stage *t*, ROIGather aggregates sampled lane features and image-level contextual information for each candidate lane. The output feature of the *i*-th candidate lane is denoted as $f_{i}^{\left(t\right)}\in R^{D}$, where i=1,…,M. The lane-level features of all candidates are stacked row-wise as

(8) $$F^{\left(t\right)}=\left[f_{1}^{(t)⊤};f_{2}^{(t)⊤};\ldots ;f_{M}^{(t)⊤}\right]\in R^{M\times D}.$$

These compact lane-level features serve as node representations for subsequent topology-aware relational reasoning.

Each lane candidate is also associated with its current geometric prediction. Following the CLRNet-style lane representation, the geometry of the *i*-th candidate lane is written as

(9) $$g_{i}^{\left(t\right)}=\left[s_{i}^{-},s_{i}^{+},y_{0,i},x_{0,i},\theta_{i},l_{i},x_{i,0},x_{i,1},\ldots ,x_{i,N-1}\right]\in R^{6+N},$$

where $s_{i}^{-}$ and $s_{i}^{+}$ are the background and foreground classification scores, $\left(x_{0,i},y_{0,i}\right)$ denotes the starting position, $\theta_{i}$ denotes the normalized orientation angle, $l_{i}$ denotes the predicted lane length, and ${\left\{x_{i,n}\right\}}_{n=0}^{N-1}$ are the normalized lateral coordinates sampled along predefined vertical positions. Only the geometric terms are used to construct the relative geometry vector.

**Relative Geometric Encoding:** To make the relation modeling sensitive to lane topology, TPR explicitly encodes the pairwise geometric configuration between lane candidates. For each ordered lane pair (i,j), we construct a relative geometry vector describing lane *j* with respect to lane *i*:

(10) $$\begin{array}{cc} \phi_{ij}^{\left(t\right)}=[ & \Delta x_{0,ij},\;\Delta y_{0,ij},\;\Delta \theta_{ij},\;\sin\left(\pi \Delta \theta_{ij}\right),\;\cos\left(\pi \Delta \theta_{ij}\right),\;\Delta l_{ij},\\ \; & {\bar{\Delta x}}_{ij},\;{\bar{\left|\Delta x\right|}}_{ij},\;\Delta x_{ij}^{\mathrm{bot}},\;\Delta x_{ij}^{\mathrm{top}},\;o_{ij}]\in R^{11}. \end{array}$$

Specifically, the relative start-point, orientation, and length terms are defined as

(11) $$\Delta x_{0,ij}=x_{0,j}-x_{0,i},\;\Delta y_{0,ij}=y_{0,j}-y_{0,i},$$

(12) $$\Delta \theta_{ij}=\theta_{j}-\theta_{i},\;\Delta l_{ij}=\frac{l_{j}-l_{i}}{N}.$$

Here, $\Delta \theta_{ij}$ is computed in the normalized angle space, and therefore $\sin(\pi \Delta \theta_{ij})$ and $\cos(\pi \Delta \theta_{ij})$ are used to provide a periodic encoding of the relative orientation.

Since not all sampled lateral coordinates are valid for every candidate lane, we define the common valid sampled-point set of lanes *i* and *j* as

(13) $$\Omega_{ij}=\left\{n|0\leq x_{i,n}\leq 1,\;0\leq x_{j,n}\leq 1,\;n=0,\ldots ,N-1\right\}.$$

Let $c_{ij}=\max(|\Omega_{ij}\left|,1\right)$ denote the valid count used for normalization. The mean signed and absolute lateral displacements are then computed as

(14) $${\bar{\Delta x}}_{ij}=\frac{1}{c_{ij}}\sum_{n\in \Omega_{ij}}\left(x_{j,n}-x_{i,n}\right),$$

(15) $${\bar{\left|\Delta x\right|}}_{ij}=\frac{1}{c_{ij}}\sum_{n\in \Omega_{ij}}\left|x_{j,n}-x_{i,n}\right|.$$

The overlap ratio is defined as the proportion of commonly valid sampled points:

(16) $$o_{ij}=\frac{|\Omega_{ij}|}{N}.$$

In the adopted lane representation, the sampled coordinates are ordered from bottom to top. Therefore, the bottom and top lateral displacements are defined as

(17) $$\Delta x_{ij}^{\mathrm{bot}}=x_{j,0}-x_{i,0},\;\Delta x_{ij}^{\mathrm{top}}=x_{j,N-1}-x_{i,N-1}.$$

The relative geometry vector is projected by a lightweight relation encoder:

(18) $$e_{ij}^{\left(t\right)}=E_{\mathrm{rel}}\left(\phi_{ij}^{\left(t\right)}\right)\in R^{D_{r}},$$

where $E_{\mathrm{rel}}\left(\cdot \right)$ is implemented by a multi-layer perceptron, and $D_{r}$ denotes the dimension of the relation embedding.

The relation embedding is further projected into two complementary geometry-aware terms:

(19) $$\beta_{ij}^{\left(t\right)}=W_{b}e_{ij}^{\left(t\right)}\in R^{H_{a}},\;u_{ij}^{\left(t\right)}=W_{u}e_{ij}^{\left(t\right)}\in R^{D},$$

where $W_{b}\in R^{H_{a}\times D_{r}}$ and $W_{u}\in R^{D\times D_{r}}$ are learnable linear projection matrices. The vector $\beta_{ij}^{\left(t\right)}$ provides head-specific relative geometry biases for attention computation, and its *h*-th element is denoted as $\beta_{ij}^{h,(t)}$. The vector $u_{ij}^{\left(t\right)}$ provides a geometry-aware relation message and is reshaped into ${\left\{u_{ij}^{h,(t)}\right\}}_{h=1}^{H_{a}}$, where $u_{ij}^{h,(t)}\in R^{d}$.

**Topology-aware Lane Affinity Construction.** Given the lane-level feature matrix $F^{\left(t\right)}$, TPR first computes query, key, and value projections:

(20) $$Q^{\left(t\right)}=F^{\left(t\right)}W_{Q},\;K^{\left(t\right)}=F^{\left(t\right)}W_{K},\;V^{\left(t\right)}=F^{\left(t\right)}W_{V},$$

where $W_{Q},W_{K},W_{V}\in R^{D\times D}$ are learnable projection matrices. The projected features are reshaped into $H_{a}$ attention heads, yielding $q_{i}^{h},k_{i}^{h},v_{i}^{h}\in R^{d}$ for the *h*-th head.

The attention logit from lane *i* to lane *j* in the *h*-th head is computed by combining feature similarity with the relative geometry bias:

(21) $$s_{ij}^{h}=\frac{{\left(q_{i}^{h}\right)}^{⊤}k_{j}^{h}}{\sqrt{d}}+\beta_{ij}^{h}.$$

The normalized topology-aware lane affinity is obtained by applying softmax over all source lanes:

(22) $$a_{ij}^{h}=\frac{\exp(s_{ij}^{h})}{\sum_{k=1}^{M}\exp\left(s_{ik}^{h}\right)}.$$

The resulting affinity matrix $A^{h,(t)}=\left[a_{ij}^{h}\right]\in R^{M\times M}$ indicates how much information lane *i* aggregates from lane *j* under both visual feature similarity and relative geometric constraints.

**Relational Aggregation.** Based on the learned topology-aware affinity matrix, TPR aggregates both visual content messages and geometry-aware relation messages for each lane candidate:

(23) $$m_{i}^{h}=\sum_{j=1}^{M}a_{ij}^{h}\left(v_{j}^{h}+u_{ij}^{h}\right),$$

where $v_{j}^{h}$ denotes the visual content message from lane *j*, and $u_{ij}^{h}$ denotes the geometry-aware message that describes lane *j* relative to lane *i*.

Messages from all attention heads are concatenated and projected back to the original feature space:

(24) $$m_{i}=W_{O}{\mathrm{Concat}}_{h=1}^{H_{a}}\left(m_{i}^{h}\right)\in R^{D},$$

where $W_{O}\in R^{D\times H_{a}d}$ is a learnable output projection matrix, and $H_{a}d=D$.

Finally, the topology-enhanced lane feature is obtained through residual updates, layer normalization, and a feed-forward network:

(25) $${\hat{f}}_{i}^{\left(t\right)}=\mathrm{LN}\left(f_{i}^{\left(t\right)}+m_{i}\right),$$

(26) $${\tilde{f}}_{i}^{\left(t\right)}=\mathrm{LN}\left({\hat{f}}_{i}^{\left(t\right)}+\mathrm{FFN}\left({\hat{f}}_{i}^{\left(t\right)}\right)\right).$$

The output features ${\left\{{\tilde{f}}_{i}^{\left(t\right)}\right\}}_{i=1}^{M}$ are topology-enhanced lane representations. Compared with the original lane features, they preserve local appearance cues while incorporating relational information from other candidate lanes. At each refinement stage, these features are fed into the subsequent prediction head to generate classification scores and geometric residuals. Since TPR is applied at every refinement stage, inter-lane relationships are progressively updated as lane geometry becomes more accurate.

### 2.4. Topology-Decoupled Head

In lane detection, classification and geometric regression emphasize different optimization objectives. Classification focuses on distinguishing foreground lane candidates from background proposals, whereas geometric regression requires accurate localization of lane start positions, orientations, lengths, and sampled lateral coordinates. Optimizing these objectives through a shared prediction pathway may introduce task interference, especially when visually ambiguous lane candidates require different discriminative and localization cues.

To alleviate this issue, we introduce a TDH to separate lane classification and geometric regression at the prediction level. Instead of applying the decoupled prediction only after the final refinement stage, TDH is used at each refinement stage. Let ${\tilde{f}}_{i}^{\left(t\right)}\in R^{D}$ denote the topology-enhanced feature of the *i*-th lane candidate at refinement stage *t*, where *D* is the feature dimension. TDH first initializes two branch-specific feature transformations from the same topology-enhanced lane feature:

(27) $${\tilde{f}}_{i}^{\left(t\right)}⟶\left\{\begin{array}{c} h_{i,\mathrm{geo}}^{\left(t\right)}={\mathrm{MLP}}_{\mathrm{geo}}\left({\tilde{f}}_{i}^{\left(t\right)}\right),\\ h_{i,\mathrm{cls}}^{\left(t\right)}={\mathrm{MLP}}_{\mathrm{cls}}\left({\tilde{f}}_{i}^{\left(t\right)}\right), \end{array}\right.$$

where $h_{i,\mathrm{geo}}^{\left(t\right)}$ and $h_{i,\mathrm{cls}}^{\left(t\right)}$ are the intermediate features of the geometry and classification branches, respectively. Each branch consists of stacked fully connected layers with nonlinear activations, allowing the two tasks to learn task-specific representations while still sharing the topology-aware lane feature as input.

The geometry branch predicts a regression vector $r_{i}^{\left(t\right)}\in R^{N+4}$:

(28) $$r_{i}^{\left(t\right)}={\mathrm{FC}}_{\mathrm{geo}}\left(h_{i,\mathrm{geo}}^{\left(t\right)}\right)\in R^{N+4}.$$

Specifically, the regression vector is written as

(29) $$r_{i}^{\left(t\right)}=\left[\Delta y_{0,i}^{\left(t\right)},\Delta x_{0,i}^{\left(t\right)},\Delta \theta_{i}^{\left(t\right)},l_{i}^{\left(t\right)},\delta x_{i,0}^{\left(t\right)},\delta x_{i,1}^{\left(t\right)},\ldots ,\delta x_{i,N-1}^{\left(t\right)}\right],$$

where $\Delta y_{0,i}^{\left(t\right)}$, $\Delta x_{0,i}^{\left(t\right)}$, and $\Delta \theta_{i}^{\left(t\right)}$ are residual updates for the start position and orientation of the current lane prior, $l_{i}^{\left(t\right)}$ denotes the predicted lane length, and ${\left\{\delta x_{i,n}^{\left(t\right)}\right\}}_{n=0}^{N-1}$ are the point-wise lateral offsets for the *N* sampled positions.

Given the lane prior from the previous stage, the start position and orientation are updated as

(30) $$\begin{array}{cc} y_{0,i}^{\left(t\right)} & =y_{0,i}^{\left(t-1\right)}+\Delta y_{0,i}^{\left(t\right)},\\ x_{0,i}^{\left(t\right)} & =x_{0,i}^{\left(t-1\right)}+\Delta x_{0,i}^{\left(t\right)},\\ \theta_{i}^{\left(t\right)} & =\theta_{i}^{\left(t-1\right)}+\Delta \theta_{i}^{\left(t\right)}. \end{array}$$

The updated prior parameters define a coarse lane hypothesis ${\bar{x}}_{i,n}^{\left(t\right)}$ at each sampled vertical position $y_{n}$. The final lateral coordinate is then obtained by adding the predicted point-wise offset:

(31) $${\hat{x}}_{i,n}^{\left(t\right)}={\bar{x}}_{i,n}^{\left(t\right)}+\delta x_{i,n}^{\left(t\right)},\;n=0,1,\ldots ,N-1.$$

In this way, TDH preserves the iterative refinement mechanism of the baseline lane representation while assigning geometry-specific parameters to an independent regression pathway.

The classification branch predicts a two-dimensional logit vector:

(32) $$c_{i}^{\left(t\right)}={\mathrm{FC}}_{\mathrm{cls}}\left(h_{i,\mathrm{cls}}^{\left(t\right)}\right)\in R^{2},$$

where the two logits correspond to the background and foreground lane classes. During inference, the foreground confidence of the *i*-th lane candidate is obtained by applying a softmax operation:

(33) $$p_{i,\mathrm{fg}}^{\left(t\right)}=\frac{\exp(c_{i,\mathrm{fg}}^{\left(t\right)})}{\exp\left(c_{i,\mathrm{bg}}^{\left(t\right)}\right)+\exp\left(c_{i,\mathrm{fg}}^{\left(t\right)}\right)}.$$

Low-confidence candidates are filtered according to this foreground probability before non-maximum suppression.

To further encourage feature-level disentanglement between the two prediction tasks, we introduce an orthogonality regularization term on the branch-specific intermediate features. Let $H_{\mathrm{geo}}^{\left(T\right)}\in R^{B\times M\times d}$ and $H_{\mathrm{cls}}^{\left(T\right)}\in R^{B\times M\times d}$ denote the final-stage geometry and classification branch features, respectively, where *B* is the batch size, *M* is the number of lane candidates, and *d* is the hidden dimension of each branch. For the *b*-th image in a mini-batch, the cross-branch correlation matrix is computed as

(34) $$C^{b}={\left(H_{\mathrm{geo}}^{b,T}\right)}^{⊤}H_{\mathrm{cls}}^{b,T}\in R^{d\times d}.$$

The orthogonality loss is defined as a normalized squared correlation penalty:

(35) $$L_{\mathrm{orth}}=\frac{1}{Z}\cdot \frac{1}{Bd^{2}}\sum_{b=1}^{B}{∥C^{b}∥}_{F}^{2},$$

where ${∥\cdot ∥}_{F}$ denotes the Frobenius norm and *Z* is a normalization factor used to stabilize the loss scale. In implementation, *Z* is set according to the number of lane candidates and the hidden dimension. This regularization suppresses excessive correlation between the geometry and classification feature subspaces, thereby reducing task interference without changing the inference pipeline.

Overall, TDH provides a lightweight prediction-level decoupling mechanism. The geometry branch focuses on lane shape refinement and coordinate localization, while the classification branch focuses on foreground confidence estimation. Combined with the topology-enhanced features provided by TPR, the decoupled design improves optimization stability and helps produce more reliable lane predictions.

### 2.5. Topology Consistency Loss

Standard geometric losses supervise lane candidates mainly through point-wise regression or lane-level overlap, but they do not explicitly penalize structurally implausible predictions such as local oscillations, lane crossings, or inconsistent relative ordering. To improve the structural coherence of predicted lanes, we introduce a TCL, which imposes both intra-lane smoothness and inter-lane ordering constraints.

During training, TCL is applied only to matched positive lane candidates after dynamic label assignment. This avoids imposing structural constraints on background proposals. The predicted lateral coordinates are first decoded into the image coordinate system. Therefore, unless otherwise specified, the coordinates used in this section are measured in pixels rather than in normalized coordinates.

Let ${\hat{P}}_{i}={\left\{\left({\hat{x}}_{i,n},y_{n}\right)\right\}}_{n=0}^{N-1}$ denote the *i*-th matched positive predicted lane, where ${\hat{x}}_{i,n}$ is the decoded lateral coordinate at the sampled vertical position $y_{n}$, and *N* is the number of sampled points. Since not all sampled points are valid for every predicted lane, we define a validity mask

(36) $$m_{i,n}=I\left(0\leq {\hat{x}}_{i,n}<W\right),$$

where *W* is the image width and I(·) is the indicator function.

#### 2.5.1. Smoothness Loss

Lane markings are continuous curvilinear structures, and their lateral coordinates should vary smoothly along the vertical direction. To suppress high-frequency oscillations, we penalize the second-order finite difference of the predicted lateral coordinates. For a valid triplet of consecutive sampled points, the second-order difference is computed as

(37) $$d_{i,n}={\hat{x}}_{i,n+1}-2{\hat{x}}_{i,n}+{\hat{x}}_{i,n-1},\;n=1,\ldots ,N-2.$$

The corresponding valid-triplet mask is defined as

(38) $$m_{i,n}^{\mathrm{sm}}=m_{i,n-1}m_{i,n}m_{i,n+1}.$$

The smoothness loss is then formulated as

(39) $$L_{\mathrm{smooth}}=\frac{\sum_{i}\sum_{n=1}^{N-2}m_{i,n}^{\mathrm{sm}}d_{i,n}^{2}}{\sum_{i}\sum_{n=1}^{N-2}m_{i,n}^{\mathrm{sm}}+\epsilon },$$

where ϵ is a small constant for numerical stability. This loss is computed only over valid triplets whose three lateral coordinates fall inside the image boundary.

#### 2.5.2. Order Loss

In real road scenes, adjacent lane markings usually preserve a consistent left-to-right spatial order. However, predicted lane candidates are not inherently ordered. Therefore, before applying the ordering constraint, we first sort valid predicted lanes according to their mean lateral positions. For the *i*-th matched positive lane, its mean lateral coordinate is computed as

(40) $${\bar{x}}_{i}=\frac{\sum_{n=0}^{N-1}m_{i,n}{\hat{x}}_{i,n}}{\sum_{n=0}^{N-1}m_{i,n}+\epsilon }.$$

Let π(·) denote the permutation that sorts the valid lanes in ascending order of ${\bar{x}}_{i}$, from left to right. The ordering constraint is imposed only on adjacent lanes in this sorted sequence.

For two adjacent sorted lanes π(i) and π(i+1), the common valid mask at the *n*-th sampled position is defined as

(41) $$m_{i,n}^{\mathrm{ord}}=m_{\pi (i),n}m_{\pi (i+1),n}.$$

The lateral separation between the two adjacent lanes is

(42) $$s_{i,n}={\hat{x}}_{\pi (i+1),n}-{\hat{x}}_{\pi (i),n}.$$

The order loss penalizes violations of a minimum lateral separation margin:

(43) $$L_{\mathrm{order}}=\frac{\sum_{i}\sum_{n=0}^{N-1}m_{i,n}^{\mathrm{ord}}\max\left(0,\tau -s_{i,n}\right)}{\sum_{i}\sum_{n=0}^{N-1}m_{i,n}^{\mathrm{ord}}+\epsilon },$$

where τ is a pixel-level margin. This formulation prevents unnatural lane crossings and overly close adjacent predictions, while avoiding constraints on invalid or non-overlapping sampled positions.

The proposed topology consistency loss is defined as

(44) $$L_{\mathrm{TCL}}=L_{\mathrm{smooth}}+\lambda_{\mathrm{order}}L_{\mathrm{order}},$$

where $\lambda_{\mathrm{order}}$ balances the contribution of the ordering constraint.

#### 2.5.3. Line IoU Loss

In addition to TCL, we retain the Line IoU (LIoU) loss as a complementary lane-level geometric supervision term. Unlike point-wise regression losses, LIoU treats each lane as a holistic geometric entity and measures the overlap between the predicted lane and its matched ground-truth lane.

Given a predicted lane ${\left\{{\hat{x}}_{n}\right\}}_{n=0}^{N-1}$ and its matched ground-truth lane ${\left\{x_{n}^{g}\right\}}_{n=0}^{N-1}$, each sampled point is extended to a horizontal line segment with half-width *e*. The per-point intersection and union are defined as

(45) $$d_{n}^{O}=\max\left(0,\min\left({\hat{x}}_{n}+e,x_{n}^{g}+e\right)-\max\left({\hat{x}}_{n}-e,x_{n}^{g}-e\right)\right),$$

and

(46) $$d_{n}^{U}=\max\left({\hat{x}}_{n}+e,x_{n}^{g}+e\right)-\min\left({\hat{x}}_{n}-e,x_{n}^{g}-e\right).$$

The LIoU loss is then computed as

(47) $$L_{\mathrm{LIoU}}=1-\frac{\sum_{n=0}^{N-1}d_{n}^{O}}{\sum_{n=0}^{N-1}d_{n}^{U}+\epsilon }.$$

In our implementation, the half-width parameter is set to e=15 pixels, which provides a stable tolerance for lane-level geometric matching.

Compared with LIoU, which focuses on the global overlap between predicted and ground-truth lanes, TCL explicitly regularizes the internal smoothness of each predicted lane and the spatial ordering among adjacent lanes. Therefore, the two losses are complementary: LIoU improves lane-level geometric alignment, while TCL encourages structurally coherent lane predictions.

### 2.6. Training and Inference

#### 2.6.1. Dynamic Label Assignment

During training, each ground-truth lane is assigned to a set of positive lane candidates through a dynamic label assignment strategy. The assignment is performed independently at each refinement stage, so that intermediate lane predictions can also receive direct supervision.

For each predicted lane candidate and ground-truth lane pair, we compute an assignment cost that combines classification confidence and geometric similarity. Let $C_{\mathrm{cls}}$ denote the focal-loss-based classification cost. To measure geometric consistency, we consider three complementary terms: the average lateral distance between sampled lane points, the start-point distance, and the orientation difference.

Given a predicted lane $p_{i}$ and a ground-truth lane $g_{j}$, let $D_{\mathrm{dis}}\left(i,j\right)$ denote the average lateral point distance computed over valid ground-truth sampled positions. Let $D_{xy}\left(i,j\right)$ denote the Euclidean distance between their start points, and let $D_{\theta }\left(i,j\right)$ denote the orientation difference. These distance terms are normalized and converted into similarity scores:

(48) $$S_{\mathrm{dis}}\left(i,j\right)=1-\frac{D_{\mathrm{dis}}\left(i,j\right)}{\max_{i,j}D_{\mathrm{dis}}\left(i,j\right)}+\epsilon ,$$

(49) $$S_{xy}\left(i,j\right)=1-\frac{D_{xy}\left(i,j\right)}{\max_{i,j}D_{xy}\left(i,j\right)}+\epsilon ,$$

and

(50) $$S_{\theta }\left(i,j\right)=1-\frac{D_{\theta }\left(i,j\right)}{\max_{i,j}D_{\theta }\left(i,j\right)}+\epsilon ,$$

where ϵ is a small constant for numerical stability. The overall geometric similarity is then defined as

(51) $$S_{\mathrm{geo}}\left(i,j\right)={\left(S_{\mathrm{dis}}\left(i,j\right)\cdot S_{xy}\left(i,j\right)\cdot S_{\theta }\left(i,j\right)\right)}^{2}.$$

The final assignment cost is formulated as

(52) $$C_{\mathrm{assign}}\left(i,j\right)=w_{\mathrm{cls}}C_{\mathrm{cls}}\left(i,j\right)-w_{\mathrm{geo}}S_{\mathrm{geo}}\left(i,j\right),$$

where $w_{\mathrm{cls}}$ and $w_{\mathrm{geo}}$ balance the classification cost and geometric similarity. A lower assignment cost indicates that the predicted candidate is more suitable for the corresponding ground-truth lane.

Instead of assigning a fixed number of positive candidates to each ground-truth lane, we adopt a dynamic-*k* strategy. We first compute the pairwise LIoU between predicted lanes and ground-truth lanes. For the *j*-th ground-truth lane, the number of positive candidates is determined by the sum of the top-*q* LIoU values:

(53) $$k_{j}=\max\left(1,\left\lfloor \sum_{\ell =1}^{q}{\mathrm{TopIoU}}_{\ell ,j}\right\rfloor \right),$$

where q=4 in our implementation. For each ground-truth lane, the $k_{j}$ predicted candidates with the lowest assignment costs are selected as positive samples. If one predicted candidate is assigned to multiple ground-truth lanes, only the assignment with the lowest cost is retained.

#### 2.6.2. Overall Training Objective

The proposed network is supervised by classification, prior-parameter regression, lane-level geometric alignment, auxiliary segmentation, topology consistency, and branch orthogonality losses. Since TPDNet follows an iterative refinement pipeline, the classification, prior-parameter regression, LIoU, and topology consistency losses are computed at each refinement stage and averaged over all stages.

At refinement stage *t*, the stage-wise detection loss is written as

(54) $$L_{\mathrm{stage}}^{\left(t\right)}=w_{\mathrm{cls}}L_{\mathrm{cls}}^{\left(t\right)}+w_{\mathrm{xytl}}L_{\mathrm{xytl}}^{\left(t\right)}+w_{\mathrm{LIoU}}L_{\mathrm{LIoU}}^{\left(t\right)}+w_{\mathrm{TCL}}L_{\mathrm{TCL}}^{\left(t\right)}.$$

Here, $L_{\mathrm{cls}}^{\left(t\right)}$ is the focal classification loss for foreground/background prediction, and $L_{\mathrm{xytl}}^{\left(t\right)}$ is the smooth-$L_{1}$ loss applied to the lane prior parameters, including start position, orientation, and length. $L_{\mathrm{LIoU}}^{\left(t\right)}$ measures lane-level geometric overlap, while $L_{\mathrm{TCL}}^{\left(t\right)}$ imposes smoothness and ordering constraints on matched positive lanes.

The stage-wise losses are averaged across all *T* refinement stages:

(55) $$L_{\det}=\frac{1}{T}\sum_{t=1}^{T}L_{\mathrm{stage}}^{\left(t\right)}.$$

In addition, an auxiliary segmentation loss $L_{\mathrm{seg}}$ is applied to the segmentation branch to strengthen dense visual supervision. The orthogonality regularization $L_{\mathrm{orth}}$ is computed using the final-stage geometry and classification branch features, encouraging feature-level disentanglement between the two prediction tasks.

The complete training objective is therefore

(56) $$L_{\mathrm{total}}=L_{\det}+w_{\mathrm{seg}}L_{\mathrm{seg}}+w_{\mathrm{orth}}L_{\mathrm{orth}}.$$

Equivalently, expanding the stage-wise detection terms gives

(57) $$L_{\mathrm{total}}=\frac{1}{T}\sum_{t=1}^{T}\left(w_{\mathrm{cls}}L_{\mathrm{cls}}^{\left(t\right)}+w_{\mathrm{xytl}}L_{\mathrm{xytl}}^{\left(t\right)}+w_{\mathrm{LIoU}}L_{\mathrm{LIoU}}^{\left(t\right)}+w_{\mathrm{TCL}}L_{\mathrm{TCL}}^{\left(t\right)}\right)+w_{\mathrm{seg}}L_{\mathrm{seg}}+w_{\mathrm{orth}}L_{\mathrm{orth}}.$$

All balancing weights are treated as hyperparameters and are set according to the validation performance.

#### 2.6.3. Inference

During inference, only the predictions from the final refinement stage are used. For each lane candidate, the classification branch outputs a two-dimensional logit vector corresponding to the background and foreground classes. The foreground confidence is obtained by applying a softmax operation:

(58) $$p_{\mathrm{fg}}=\frac{\exp(c_{\mathrm{fg}})}{\exp\left(c_{\mathrm{bg}}\right)+\exp\left(c_{\mathrm{fg}}\right)}.$$

Candidates with foreground confidence lower than a predefined threshold are discarded.

After confidence filtering, Line-IoU-based non-maximum suppression is applied to remove redundant lane predictions. The remaining candidates are decoded into lane point sequences according to their predicted start position, length, and sampled lateral coordinates. Since the final lane geometry is decoded from the regression branch, the decoupled prediction design reduces direct interference from the classification branch during lane localization.

The final output is a set of lane instances:

(59) $$Y=\left\{{\hat{P}}_{i}|p_{i,\mathrm{fg}}\geq \eta ,\;i\in I_{\mathrm{NMS}}\right\},$$

where η is the confidence threshold and $I_{\mathrm{NMS}}$ denotes the set of lane candidates retained after non-maximum suppression.

## 3. Results

### 3.1. Datasets

To evaluate the effectiveness and generalization capability of the proposed TPDNet, we conduct experiments on three widely used lane detection benchmarks: CULane, TuSimple, and CurveLanes. These datasets cover different driving scenarios, ranging from regular highway scenes to complex urban roads and highly curved lane layouts.

- **CULane** is a large-scale lane detection dataset collected in complex urban driving environments, containing approximately 133,000 images. It covers nine challenging scenarios, including *Normal*, *Crowded*, *Dazzle light*, *Shadow*, *No line*, *Arrow*, *Curve*, *Cross*, and *Night*. Following the official protocol, the dataset is split into 88,880 training images, 9675 validation images, and 34,680 testing images. Due to frequent occlusions, illumination changes, worn lane markings, and complex road layouts, CULane provides a challenging benchmark for evaluating robustness in real-world driving scenarios.
- **TuSimple** mainly focuses on highway driving scenes under relatively stable illumination conditions. It consists of 6408 training images and 2782 testing images. Compared with CULane, TuSimple contains more regular lane structures and cleaner road scenes, making it suitable for evaluating the geometric localization accuracy of lane detection methods.
- **CurveLanes** is designed for lane detection in complex curved road scenes. It contains 150,000 high-resolution images, including 100,000 training images, 20,000 validation images, and 30,000 testing images. Since the test annotations are not publicly available, we follow common practice in existing CurveLanes evaluations [19,24] and report results on the validation split. The dataset contains abundant sharp curves, dense lane structures, and multi-lane bifurcations, making it particularly suitable for evaluating topology-aware lane reasoning.

**Data Preprocessing.** For all datasets, input images are resized to a fixed resolution, e.g., 800×320, to balance computational efficiency and feature resolution. Ground-truth lane annotations are converted into sampled point sequences according to the lane representation described in Section 2.2. During training, standard data augmentation strategies, including random horizontal flipping, rotation, and brightness jittering, are applied to improve generalization.

### 3.2. Implementation Details and Evaluation Metrics

**Implementation Details.** The proposed framework is implemented in PyTorch 2.0 and trained on an NVIDIA RTX 3090 GPU (Nvidia Corporation, Santa, Clara, CA, USA). We use ResNet-18, ResNet-34, and ResNet-101 as backbone networks, equipped with a Feature Pyramid Network (FPN) for multi-scale feature extraction. The model is optimized using AdamW with an initial learning rate of $1\times 10^{-4}$ and a cosine annealing learning-rate schedule. The batch size is set to 8, and the model is trained for 30 epochs. For data augmentation, we apply random horizontal flipping, rotation, and brightness jittering.

**Evaluation Metrics.** For fair comparison, all reported results are evaluated following the official evaluation protocols of the corresponding datasets. On CULane, a predicted lane is considered a true positive if its intersection-over-union with a ground-truth lane is greater than 0.5. Otherwise, it is counted as a false positive, while unmatched ground-truth lanes are counted as false negatives. Precision, recall, and F1 score are computed as

(60) $$\mathrm{Precision}=\frac{\mathrm{TP}}{\mathrm{TP}+\mathrm{FP}},\;\mathrm{Recall}=\frac{\mathrm{TP}}{\mathrm{TP}+\mathrm{FN}},$$

(61) $$F1=\frac{2\cdot \mathrm{Precision}\cdot \mathrm{Recall}}{\mathrm{Precision}+\mathrm{Recall}}.$$

For TuSimple, we report both the official accuracy metric and the F1 score. For CurveLanes, we report mF1, F1@50, F1@75, precision, recall, FPS, and computational cost following the commonly used evaluation setting. For FPS reporting, we use the same end-to-end inference pipeline as in deployment, including confidence filtering and Line-IoU-based non-maximum suppression, so the reported speed reflects the final prediction workflow rather than the raw network forward pass alone.

Because the proposed method is motivated by structural consistency, we also consider topology-aware diagnostic metrics in addition to standard F1-based scores. Let P denote the set of matched predicted lanes in an image, and let $x_{i}\left(y\right)$ be the lateral coordinate of lane *i* at vertical position *y*. We evaluate three complementary structural indicators:

- **Lane ordering accuracy (LOA)** measures the proportion of valid adjacent lane pairs whose left-to-right order is consistent across their shared vertical support. Higher LOA indicates fewer ordering inversions.
- **Smoothness error (SE)** is the average squared second-order difference in sampled lane coordinates, $\frac{1}{\left|P\right|}\sum_{i}{\mathrm{mean}}_{y}{\left(x_{i}\left(y+1\right)-2x_{i}\left(y\right)+x_{i}\left(y-1\right)\right)}^{2}$. Lower SE indicates smoother lane trajectories.
- **Lane crossing rate (LCR)** measures the percentage of valid adjacent lane pairs that exhibit at least one left–right sign reversal over their shared support. Lower LCR indicates fewer structurally implausible crossings.

These diagnostic metrics are not intended to replace official benchmark metrics, but to directly examine the structural properties targeted by TPR and TCL.

### 3.3. Comparative Analysis

To evaluate the effectiveness of TPDNet, we first conduct a comparative analysis on the CULane dataset, which is one of the most widely used benchmarks for urban-scene lane detection. We compare TPDNet with representative lane detection methods, including SCNN, RESA, UFLD, LaneATT, CondLane, CLRNet, and CLRerNet.

The quantitative results across the nine challenging CULane scenarios are summarized in Table 1. TPDNet achieves highly competitive overall performance. With ResNet-101, TPDNet obtains an F1@50 score of 81.46, which is slightly higher than the strongest compared method. Compared with existing methods, the improvement is more noticeable in topology-sensitive and visually challenging scenarios, such as *Dazzle light*, *Shadow*, *No line*, *Arrow*, *Curve*, and *Cross*.

These results suggest that explicitly modeling inter-lane structural relationships is beneficial for robust lane detection under degraded visual conditions. In particular, the improvements in *Curve* and *Cross* scenarios indicate that topology-aware reasoning helps preserve lane continuity and relative spatial ordering when the road layout becomes complex. The performance in *Dazzle light* and *Shadow* scenarios further shows that relational reasoning can provide useful structural cues when local lane appearance is unreliable.

To further evaluate the generalization capability of TPDNet, we report its performance on TuSimple and CurveLanes in Table 2 and Table 3, respectively.

On TuSimple, TPDNet achieves a strong F1 score among the compared methods while maintaining competitive accuracy. Since TuSimple contains relatively regular highway scenes, the strong F1 score indicates that the proposed method can preserve accurate lane localization in structured driving environments. Meanwhile, the low false-positive and false-negative rates show that TPDNet achieves a balanced trade-off between precision and recall.

The advantage of TPDNet is more evident on CurveLanes, as shown in Table 3. With ResNet-34, TPDNet achieves an mF1 score of 58.74, outperforming the strongest compared baseline by 4.98 points. This improvement is particularly meaningful because CurveLanes contains abundant curved lanes, dense lane layouts, and complex multi-lane structures. The strong performance gain suggests that geometry-aware inter-lane reasoning helps the model maintain coherent lane structures when the road topology is highly non-linear.

Overall, the results across the three benchmarks demonstrate that TPDNet achieves a favorable balance between local geometric accuracy and global structural consistency. Instead of treating lane candidates as isolated instances, the proposed topology-aware design allows lane features to interact with each other, which improves robustness in challenging road scenarios.

**Figure 3:**
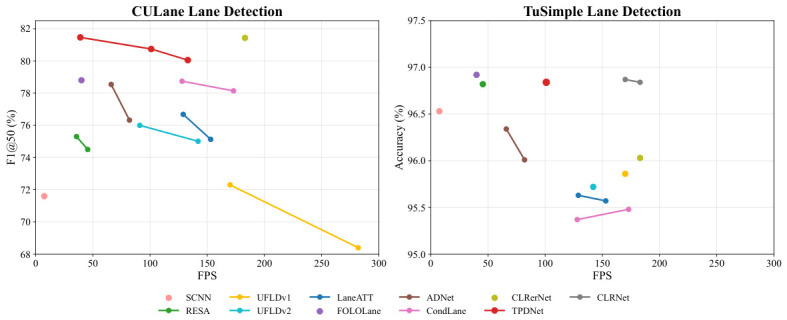
Accuracy–efficiency comparison on the CULane and TuSimple datasets. TPDNet provides a favorable balance between detection performance and inference speed under different backbone settings.

**Efficiency Analysis.** To further assess the accuracy–efficiency trade-off, we compare detection performance and inference speed, as shown in Figure 3. Lightweight variants of TPDNet maintain competitive inference speed while improving detection accuracy. The ResNet-101 variant achieves higher accuracy but introduces additional computational cost. This trend indicates that the proposed topology-aware modules can be integrated into different backbone settings, allowing a flexible trade-off between accuracy and efficiency according to practical deployment requirements.

The main additional cost of TPR comes from pairwise lane-candidate attention. For *M* lane candidates, feature dimension *D*, relation embedding dimension $D_{r}$, and *T* refinement stages, the complexity of TPR is $O(T\left(M^{2}D+M^{2}D_{r}+MD^{2}\right))$, and the dominant pairwise memory terms are the attention map of size $H_{a}M^{2}$ and the relation embedding of size $M^{2}D_{r}$. In our default setting, M=192, D=64, $D_{r}=64$, $H_{a}=4$, and T=3. Thus, the TPR module introduces a quadratic dependency on the number of lane candidates, but the absolute cost remains bounded because lane detection uses a fixed and relatively small proposal set. For deployment-oriented settings, reducing *M*, sharing relation encoders across stages, or sparsely attending to the top-*k* geometrically relevant candidates can further lower the memory and latency overhead. We leave a module-wise breakdown of parameters, latency, and memory usage as future work for a more deployment-oriented study.

### 3.4. Ablation Study

To evaluate the contribution of each proposed component, we conduct ablation experiments on CULane by progressively adding TPR, TDH, and TCL to the baseline model. The results are summarized in Table 4.

The baseline model achieves an F1@50 of 80.13. After introducing TPR, the performance increases to 80.51, yielding a gain of 0.38 points. This is the largest single improvement among the three components, indicating that explicit inter-lane relational reasoning plays an important role in improving lane detection performance. By allowing lane candidates to aggregate information from other structurally related candidates, TPR helps the model capture global road layout information that is difficult to obtain from isolated lane features.

Adding TDH further improves the F1@50 score to 80.66. This result suggests that separating geometric regression and classification into branch-specific prediction paths can reduce task interference and improve optimization stability. Since lane classification focuses on foreground–background discrimination while geometric regression emphasizes precise coordinate localization, the decoupled design allows the two tasks to learn more suitable representations.

When TCL is further incorporated, the model achieves the best F1@50 score of 80.74. Although the numerical gain from TCL is moderate, it provides an explicit structural regularization that complements the LIoU loss. While LIoU encourages the predicted lanes to align with ground-truth lanes, TCL further regularizes the internal smoothness of each lane and the relative ordering among adjacent lanes. This helps reduce structurally implausible predictions such as local oscillations and lane crossings.

The complete model achieves a total improvement of 0.61 points over the baseline. These ablation results show that TPR, TDH, and TCL contribute complementary benefits: TPR enhances feature-level relational reasoning, TDH improves prediction-level task decoupling, and TCL strengthens supervision-level structural consistency.

### 3.5. Structural Consistency, Statistical Stability, and Sensitivity Analysis

To directly examine whether the proposed topology-aware components improve structural quality, we further evaluate the ablation variants using the topology-aware metrics defined in Section 3.2. As shown in Table 5, adding TPR and TCL is expected to improve lane ordering accuracy and reduce smoothness error and crossing rate, because TPR explicitly propagates inter-lane geometric context and TCL directly regularizes intra-lane smoothness and inter-lane ordering.

To assess experimental stability, we repeat the main CULane experiment with multiple random seeds and report the mean and standard deviation in Table 6. This analysis is particularly important because the overall F1 margin between the strongest methods is narrow.

We also analyze the sensitivity of TCL to the order margin τ and the order-loss weight $\lambda_{\mathrm{order}}$. Table 7 summarizes the validation performance under different settings. In practice, we select the default hyperparameters according to validation performance and use the same setting for all test-set comparisons. Although this analysis captures the main hyperparameter trend, a finer-grained decomposition of the smoothness loss, order loss, orthogonality regularization, and individual relative-geometry terms in TPR would provide an even more exhaustive picture and is left for future work.

### 3.6. Qualitative Analysis and Visualization

**Figure 4:**
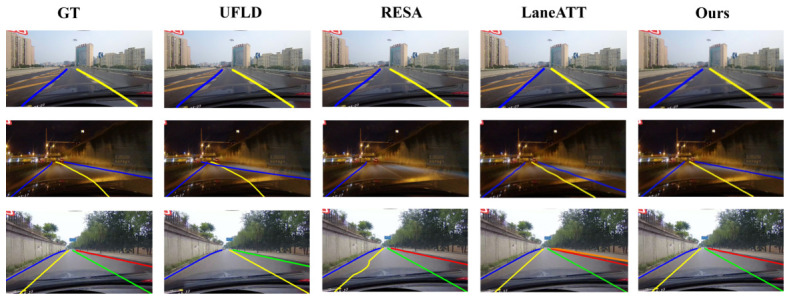
Qualitative comparison on the CULane dataset. TPDNet produces more continuous and structurally coherent lane predictions under challenging conditions.

To qualitatively analyze the behavior of TPDNet, we visualize lane detection results under challenging CULane scenarios. As shown in Figure 4, TPDNet produces more continuous and coherent lane structures compared with representative baseline methods.

In scenes with weak lane markings, illumination disturbance, or partial occlusion, competing methods may generate fragmented predictions, miss partially visible lanes, or produce unstable lane structures. In contrast, TPDNet better preserves the continuity and relative ordering of adjacent lanes. This is consistent with the design of TPR and TCL: TPR enables candidate lanes to exchange structural information through topology-aware relational reasoning, while TCL encourages smooth and ordered lane predictions during training.

These qualitative results further support the quantitative findings. The proposed topology-aware design not only improves numerical performance, but also helps generate lane predictions that are more consistent with the underlying road structure.

### 3.7. Failure Case Analysis

We further inspect failure cases in scenarios that are not fully captured by standard F1-based evaluation. Under severe occlusion, TPDNet can still infer short missing lane segments when neighboring lanes provide reliable structural cues, but long-term disappearance may lead to missed detections because the current framework does not use temporal evidence across frames. In lane merging or splitting regions, the ordering constraint in TCL may become less reliable because the true topology changes along the vertical axis; in such cases, overly strong ordering regularization can suppress valid convergence patterns. For heavily degraded or nearly invisible lane markings, the proposal-based pipeline may fail when the initial lane candidates do not sufficiently cover the true lane positions. These cases indicate that topology-aware spatial reasoning improves structural coherence, but it cannot completely replace temporal modeling, adaptive topology switching, or stronger proposal generation under extreme visual degradation.

## 4. Discussion

This work revisits lane detection from the perspective of structural consistency. Instead of treating lane candidates as independent prediction targets, TPDNet introduces topology-aware reasoning into an iterative lane refinement framework. The core idea is to improve lane detection not only by enhancing local visual features, but also by modeling the structural relationships among lane instances.

The proposed TPR plays the central role in feature-level relational reasoning. By incorporating both lane-query features and pairwise geometric encodings, TPR allows each candidate lane to aggregate information from other structurally related candidates through learned topology-aware affinities. In this way, the model can exploit inter-lane cues such as relative ordering, spatial proximity, and geometric continuity. This is particularly useful when local visual evidence is unreliable due to occlusion, shadows, dazzle light, worn markings, or complex road curvature.

In addition to feature-level reasoning, TDH and TCL provide complementary improvements at the prediction and supervision levels. TDH separates geometric regression from foreground-background classification, reducing direct task interference during lane prediction. TCL further regularizes the predicted lane structures by encouraging intra-lane smoothness and inter-lane ordering consistency. Together, these components form a multi-level topology-aware design: TPR enhances relational representation, TDH improves task-specific prediction, and TCL encourages structurally coherent outputs.

The experimental results support this design. On CULane, TPDNet achieves competitive overall performance and shows clear improvements in several challenging scenarios where structural reasoning is important, such as *Dazzle light*, *Shadow*, *No line*, *Arrow*, and *Curve*. On TuSimple, the method maintains strong geometric localization performance under relatively regular highway scenes. On CurveLanes, where curved lanes and complex multi-lane layouts are more common, TPDNet obtains a more noticeable improvement, suggesting that geometry-aware inter-lane reasoning is especially beneficial for highly non-linear road topology.

Despite these advantages, several limitations remain. First, the current framework mainly models spatial relationships among lane candidates within a single image. It does not explicitly exploit temporal consistency across video frames, which may be useful for handling motion blur, intermittent occlusion, and short-term lane disappearance. Second, although TCL improves structural regularity, its smoothness and ordering constraints are still hand-crafted regularization terms. In extreme cases involving abrupt lane merging, splitting, or heavily degraded markings, these constraints may not fully capture the underlying road topology. Third, TPDNet is still built on a proposal-based iterative refinement pipeline, and its final performance can be affected by the quality of initial lane candidates, especially when lanes are severely occluded or nearly invisible.

Future work will focus on three directions. First, we plan to investigate adaptive topology modeling mechanisms that can dynamically adjust inter-lane relationships according to scene context, rather than relying only on pairwise geometric cues. Second, incorporating temporal information into the topology-aware reasoning process may further improve robustness in dynamic driving environments. Third, more lightweight topology reasoning modules will be explored to reduce computational overhead and improve deployment efficiency. Overall, this work shows that explicitly introducing structural reasoning into lane detection is a promising direction for improving both detection accuracy and road-layout consistency.

## 5. Conclusions

This paper presented TPDNet, a topology-aware lane detection framework that introduces structural reasoning into feature representation, prediction, and supervision. The proposed TPR performs dynamic geometry-aware relational reasoning among lane candidates, TDH decouples classification and geometric regression to reduce task interference, and TCL provides complementary smoothness and ordering regularization. Experiments on CULane, TuSimple, and CurveLanes show that TPDNet achieves competitive detection accuracy and is particularly effective in challenging scenarios involving curved lanes, degraded markings, and complex lane layouts. Beyond improving standard benchmark scores, the results suggest that explicitly modeling inter-lane topology is a useful direction for producing more coherent and reliable road structure perception in autonomous driving systems.

## Figures and Tables

**Table 1:** Comparison on the CULane test set across nine scenarios. Higher is better for all metrics except *Cross-FP*, which is reported as the number of false positives (lower is better) following the official evaluation protocol. GFlops are reported when available or measured under the same input setting.

Method	Backbone	F1@50	F1@75	FPS	GFlops	Normal	Crowded	Dazzle	Shadow	No Line	Arrow	Curve	Cross	Night
SCNN [5]	VGG16	71.60	35.09	7.5	338.4	90.60	69.70	58.50	66.90	43.40	84.10	64.40	1990	66.10
RESA [6]	ResNet34	74.50	–	45.5	42.1	91.90	72.40	66.50	72.00	46.30	88.10	68.60	1896	69.80
RESA [6]	ResNet50	75.30	53.39	35.7	43.0	92.10	73.10	69.20	72.80	47.70	88.30	70.30	1503	69.90
UFLDv1 [7]	ResNet18	68.40	40.01	**282**	**8.5**	87.70	66.00	58.40	62.80	40.20	81.00	57.90	1743	62.10
UFLDv1 [7]	ResNet34	72.30	–	170	16.8	90.70	70.20	59.50	69.30	44.40	85.70	69.50	2037	66.70
UFLDv2 [25]	ResNet18	75.01	53.55	142	14.1	91.80	73.30	65.30	75.10	47.60	87.90	68.50	2075	70.70
UFLDv2 [25]	ResNet34	76.00	75.50	91	27.0	92.50	74.80	65.50	74.10	49.20	88.80	70.10	1910	70.80
LaneATT [8]	ResNet18	75.13	51.29	153	9.4	91.17	72.71	65.82	68.03	49.13	87.82	63.75	**1020**	68.58
LaneATT [8]	ResNet34	76.68	54.34	129	18.0	92.14	75.03	66.47	78.15	49.39	88.38	67.72	1330	70.72
Laneformer [26]	ResNet50	77.06	57.50	–	58.1	95.77	75.47	70.14	75.76	48.73	87.65	65.24	19	71.08
FOLOLane [27]	ERFNet	78.80	–	40	–	92.70	77.80	75.20	79.30	52.10	89.00	69.40	1569	74.50
ADNet [28]	ResNet18	76.32	56.08	82	16.2	91.23	74.54	68.61	70.73	49.01	86.22	67.21	1127	71.02
ADNet [28]	ResNet34	78.54	56.22	66	31.3	92.77	77.35	71.74	78.08	52.58	88.96	70.46	1452	74.05
CondLane [24]	ResNet18	78.14	57.42	173	11.0	92.87	75.79	70.72	80.01	52.39	89.37	72.40	1364	73.23
CondLane [24]	ResNet34	78.74	59.39	128	19.7	93.38	77.14	71.17	79.93	51.85	89.89	73.88	1387	73.92
CLRerNet [29]	DLA34	81.43	64.98	183	18.4	93.94	80.17	73.92	83.39	55.46	90.80	74.00	1540	76.22
**TPDNet (ours)**	ResNet18	80.05	61.24	133	16.2	92.56	78.37	74.08	83.02	55.10	91.08	78.02	1670	75.16
**TPDNet (ours)**	ResNet34	80.74	61.78	101	20.6	93.31	79.26	74.98	83.16	55.74	91.76	78.61	1352	75.33
**TPDNet (ours)**	ResNet101	**81.46**	62.06	39	25.1	**94.36**	79.52	**76.97**	**84.83**	**56.73**	**91.33**	**79.03**	1289	75.81

**Table 2:** Comparison with state-of-the-art lane detection methods on the TuSimple dataset. GFlops are reported when available or measured under the same input setting.

Model	Backbone	F1 ↑	Accuracy ↑	FP ↓	FN ↓	GFlops
SCNN [5]	VGG16	95.97	96.53	6.17	**1.80**	271.2
RESA [6]	ResNet34	96.93	96.82	3.63	2.48	37.1
FOLOLane [27]	ERFNet	96.59	**96.92**	4.47	2.28	39.0
UFLDv1 [7]	ResNet34	88.02	95.86	18.91	3.75	**10.1**
UFLDv2 [25]	ResNet34	88.14	95.72	18.54	3.60	21.4
ADNet [28]	ResNet18	96.90	96.01	2.91	3.29	23.3
ADNet [28]	ResNet34	97.17	96.34	2.87	2.53	30.6
CondLane [24]	ResNet18	97.01	95.48	**2.18**	3.80	17.3
CondLane [24]	ResNet34	96.98	95.37	2.20	3.82	22.1
LaneATT [8]	ResNet18	96.71	95.57	3.56	3.01	16.6
LaneATT [8]	ResNet34	96.77	95.63	3.53	2.92	18.1
CLRNet [9]	ResNet18	97.89	96.84	2.28	1.92	33.9
CLRNet [9]	ResNet34	97.82	96.87	2.27	2.08	39.1
CLRerNet [29]	ResNet34	96.72	96.03	3.26	2.57	25.4
**TPDNet (ours)**	**ResNet34**	**98.01**	96.84	2.29	1.91	20.6

**Table 3:** Comparison with state-of-the-art lane detection methods on the CurveLanes dataset.

Method	Backbone	mF1	F1@50	F1@75	Prec.@50	Recall@50	FPS	GFlops
SCNN [5]	VGG16	-	65.02	-	76.13	56.74	-	328.4
UFLDv2 [25]	Res18	49.06	80.45	54.23	81.49	79.44	79	48.0
UFLDv2 [25]	Res34	50.48	81.34	56.49	81.93	80.76	46	95.5
CondLaneNet [24]	Res18	52.50	85.09	59.04	87.75	82.58	123	10.3
CondLaneNet [24]	Res34	53.76	85.92	61.07	88.29	83.68	87	19.7
**TPDNet**	Res18	58.62	87.10	**69.22**	90.13	82.77	101	16.2
**TPDNet**	Res34	**58.74**	**87.26**	68.47	**90.31**	**87.11**	**141**	20.6

**Table 4:** Overall ablation study of TPDNet-R34 on CULane.

Baseline	+TPR	+TDH	+TCL	F1@50
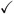				80.13
	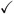			80.51 (+0.38)
	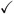	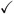		80.66 (+0.53)
	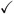	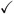	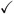	**80.74 (+0.61)**

**Table 5:** Topology-aware structural analysis on CULane. LOA denotes lane ordering accuracy, SE denotes smoothness error, and LCR denotes lane crossing rate.

Variant	F1@50 ↑	LOA ↑	SE ↓	LCR ↓
Baseline	80.13	82.45	0.452	2.14
+TPR	80.51	83.12	0.418	1.85
+TPR+TDH	80.66	83.78	0.395	1.62
+TPR+TDH+TCL	**80.74**	**84.23**	**0.371**	**1.48**

**Table 6:** Multi-run stability analysis on CULane F1@50. Values should be reported over independently trained runs with different random seeds.

Method	Seed 0	Seed 1	Seed 2	Mean ± Std.
TPDNet-R34	80.74	80.52	80.91	80.72 ± 0.20
TPDNet-R101	81.46	81.21	82.01	81.56 ± 0.41

**Table 7:** Sensitivity analysis of TCL hyperparameters on CULane validation set.

τ	$\lambda_{\mathrm{order}}$	Val F1@50
3	0.5	78.10
3	1.0	78.33
5	1.0	79.58
7	1.0	79.21
9	1.5	78.05

## Data Availability

The original contributions presented in this study are included in the article. Further inquiries can be directed to the corresponding authors.
